# Reply: Psychological Responses to Breast Density Notification: Empowerment or Anxiety?

**DOI:** 10.1111/hex.70701

**Published:** 2026-06-17

**Authors:** Emma Grundtvig Gram, Nehmat Houssami, Kirsten McCaffery, Claire Hudson, Jennifer Isautier, Brooke Nickel

**Affiliations:** ^1^ Sydney Health Literacy Lab, School of Public Health, Faculty of Medicine and Health The University of Sydney Sydney New South Wales Australia; ^2^ Center for General Practice, Department of Public Health University of Copenhagen Copenhagen Denmark; ^3^ Wiser Healthcare, School of Public Health The University of Sydney Sydney New South Wales Australia; ^4^ The Daffodil Centre, A Joint Venture With Cancer Council NSW The University of Sydney Sydney New South Wales Australia

We thank the authors for their careful reading of our article and appreciate their recognition of the study's contribution to understanding the psychosocial impact of breast density notification.

As noted, our aim was to explore what reporting psychosocial consequences entails in the context of breast density notification within a population‐based screening program. To achieve this, we purposively sampled women who had reported feeling anxious following receipt of their breast density notification alongside their screening result. This enabled an in‐depth exploration of what feeling anxious following such notification may mean and its impact. Information on several outcomes for the 2401 participants in the RCT report are already published, so this work focused specifically on the subgroup feeling anxious [1]. While we agree that the findings of this interview study do not represent the full spectrum of responses among all screening participants, this was not the objective of the study. Rather than limiting generalisability in a conventional sense, this study—and qualitative studies more generally—provides detailed insight into the experiences, interpretations, and everyday implications for women who do report feeling anxious. The women who report feeling anxious is an important subgroup when considering potential unintended consequences of communicating about breast density as part of breast screening. These insights include how women understand their risk, navigate healthcare decisions, and incorporate the information into their daily lives. These findings add to a sparse body of literature on the impact of breast density notification [2] and may even be considered when developing specific questionnaires to measure psychosocial impact of breast density notification. We also note that these considerations regarding sampling and scope are transparently acknowledged in the paper (see aims statement in introduction, section 2.1 and section 4).

We thank the authors for bringing up the point about questionnaire validation. The use of a single‐item measure in the trial [1] was a motivation for the present qualitative study to explore what self‐reported anxiousness (feeling uneasy, worried, nervous) in this context reflects in terms of lived experiences and psychosocial meaning. While standardised instruments assessing clinical states of anxiety are often used in screening settings, they are less sensitive to subtle and context‐specific psychosocial responses [3]. Our aim was not to assess clinical anxiety, but to provide a nuanced understanding of women's responses to breast density notification. This limitation is acknowledged in the paper.

We agree that there is a delicate balance between beneficial health vigilance and unnecessary worry or medicalisation following breast density notification. We also concur that self‐reported intentions do not necessarily translate into measurable health benefits, and that further research is needed to distinguish appropriate vigilance from unnecessary worry, as well as to clarify the longer‐term implications of these responses [4, 5].

We again thank the authors for their thoughtful and constructive comments, and we agree with their reflections on the importance of careful health communication, particularly in the context of screening asymptomatic populations. We appreciate their engagement with our work and their valuable perspectives on advancing the field.

## Data Availability

Data sharing not applicable to this article as no datasets were generated or analysed during the current study.
